# Interaction of estradiol and vitamin D with low skeletal muscle mass among middle-aged and elderly women

**DOI:** 10.1186/s12905-023-02646-z

**Published:** 2023-09-15

**Authors:** Jiaxing Zhang, Yalong Cheng, Chen Chen, Qingan Wang, Chan Yang, Jiangwei Qiu, Juan Li, Xiaowei Liu, Yuhong Zhang, Lan Liu, Yi Zhao

**Affiliations:** 1 School of Public Health, Ningxia Medical University, Ningxia Hui Autonomous Region, Yinchuan, China; 2 Department of Public Health, People’s Hospital of Ningxia Hui Autonomous Region, Yinchuan, Ningxia China; 3 Key Laboratory of Environmental Factors and Chronic Disease Control, Ningxia Medical University, Yinchuan, Ningxia China; 4 School of Nursing, Ningxia Medical University, Ningxia Hui Autonomous Region, Yinchuan, China

**Keywords:** Estradiol, Vitamin D, Low skeletal muscle mass, Muscle atrophy, Women

## Abstract

**Background:**

Since the connection between muscle atrophy and vitamin D and estradiol status ambiguous, this study was thus conducted to determine whether low skeletal muscle mass (SMM) in middle-aged and elderly women was affected by estradiol and vitamin D levels together.

**Methods:**

Baseline data from a sub-cohort of the China Northwest Natural Population Cohort: Ningxia Project (CNC-NX) were analyzed. Serum 25-hydroxyvitamin D (25(OH) D) and estradiol were measured by chemiluminescence immunoassay analyzer. Bivariate logistic regression and multiplicative interaction analyses were used to assess the impact of estradiol level and vitamin D status on low SMM, as well as the combined impact of estradiol and low vitamin D status on low SMM.

**Results:**

A total of 287 (9.49%) participants had low SMM, which had lower levels of estradiol and vitamin D concentration than normal SMM group. While, after adjusting the confounding variables, these correlations were maintained in estradiol Q1, Q2, Q3 and vitamin D Q1. Furthermore, the significant combined effect of the highest quartile of estradiol concentrations and non-vitamin D deficiency, and interactions between vitamin D Q1 and estradiol Q2, vitamin D Q1 and estradiol Q3, vitamin D Q2 and estradiol Q1, vitamin D Q3 and estradiol Q3 on low SMM were stably reflected (*P* for interaction < 0.05).

**Conclusions:**

Estradiol and vitamin D were interrelated with low SMM in middle-aged and elderly women. Combination of estradiol and vitamin D supplements should be encouraged for middle-aged and elderly women who are at risk of muscle atrophy or experiencing muscle atrophy.

## Introduction

Muscle atrophy is a physiological phenomenon associated with aging, characterized by a normal decline in muscle mass and function, known as sarcopenia, which is manifested in a decrease in skeletal muscle mass, muscle strength, and physical activity, and often coexists with obesity [1, 2]. Sarcopenia can further deteriorate the musculoskeletal health of the elderly by raising the risk of osteoporotic vertebral and hip fractures [3], which ultimately results in mobility restrictions, functional impairment, physical disability, and even a lower quality of life [4]. Sarcopenia not only effects very elderly individuals, but can also be widely observed in middle-aged adults [5]. Sarcopenia currently affects more than 50 million people, and by 2050, it is predicted that number will rise to more than 200 million [1]. Studies have found that skeletal muscle declines substantially from about 30 years old, with an annual loss rate of approximately 0.1–0.5%, while the rate acceleration dramatically post age 65 [6].

The reduction of skeletal muscle mass is not only linked to negative social and financial effects like hospitalization and mortality [7], also connected to comparable gender-neutral alterations, such as heightened inflammation, satellite cell senescence, decreased protein synthesis, and myocyte regeneration [8], as well as some additional gender-specific alterations brought on by a decrease in sex hormones with aging [9]. Furthermore, according to a number of studies, women have physiologically lower levels of skeletal muscle mass than men, which frequently results in sarcopenia [10, 11]. The onset of female sarcopenia appears to be more susceptible to menopause, which may be the cause of this difference between men and women [12].

Menopause is a physiological age-related condition in women that is accompanied by a drop in hormone levels, especially estradiol levels [13, 14]. Estradiol is essential for both female growth and aging, maintaining the sexual characteristics of female. Meanwhile, estradiol can also promote muscle regeneration and contribute to muscle health [1, 15]. Nevertheless, reduction of estradiol during menopause can results in musculoskeletal discomfort [16], resulting in modifications to body composition including skeletal muscle loss and fat accumulation, and even raise the danger of vitamin D deficiency, which might aggravate musculoskeletal discomfort conversely [17]. According to previous studies, the estrogen can stimulate vitamin D metabolism in postmenopausal women by increasing 1,25(OH)_2_ D levels, which could potentially explain the mechanism behind the interaction between vitamin D deficiency and estrogen regarding low skeletal muscle mass [18, 19].

Indeed, vitamin D deficiency poses a significant health challenge worldwide, especially the elderly. Numerous studies have linked a vitamin D deficiency to skeletal muscle loss and reduction in physical function [20, 21], compromising the strength of muscle in the lower limbs [22], whereas others have indicated that vitamin D has no effect on boosting muscle mass or only a weak correlation with muscle atrophy [23]. Therefore, given the association of vitamin D deficiency and muscle atrophy is still controversial, and estradiol not only plays a key role during women’s menopause but have an effect on muscle atrophy simultaneously. This study was thus conducted to determine whether low skeletal muscle mass in middle-aged and elderly women was affected by estradiol and vitamin D levels together, which may serve as an useful theoretical foundation for future combination of vitamin D and estradiol supplementation therapies.

## Materials and methods

### Study participants

The current research used cross-sectional data from the China Northwest Natural Population Cohort: Ningxia Project (CNC-NX). A prior study provided a thorough description of the research design and methods [11]. Briefly, 5300 individuals in total (2182 men and 3118 women), a representative sub-cohort consisted of 30% adults randomly selected from CNC-NX, were included in this research [19]. We excluded participants who were male, had missing data on body composition, and those who had been administered hormone therapy prior to analysis (n = 93). Besides, we filled with imputation method, based on the individuals with same demographic and laboratory data, to predict vitamin D levels for 55 participants without vitamin D concentration values. Ultimately, 3025 participants were included in the present analyses.

The present study was evaluated by the Institutional Ethics Committees (IECs) of Ningxia Medical University in accordance with the principles set forth in the Helsinki Declaration (Ethics ID 2018-012, 2020 − 689) and the written informed consent of all participants were obtained at the beginning of the investigation.

### Demographic and laboratory data

Each participant underwent all of the following procedures, which were recorded by trained researchers between March 2018 and May 2019.

#### Demographic data

After signing the informed consent form, all participants received a face-to-face questionnaire to assess demographic data [11]. The dietary intake survey included semi-quantitative food frequency (SFFQ), which made up based on a preliminary study of 24-h dietary review of the local population. No participants were found to have ever experienced eating disorders in this survey. Moreover, the dietary energy and nutrients of the participants were calculated through the Nutrition Calculator v2.8.0 (Institute of Nutrition and Food Safety, Chinese Center for Disease Control and Prevention, Beijing, China).

#### Anthropometric measurements

All participants were instructed to wear light clothes, no shoes and were forbidden to eat for 12 h, and abstain from alcohol, caffeine and strenuous activity for at least 24 h. Two measurements of weight and height were taken for each individual, rounded to the nearest 0.1 kg and 0.1 cm, respectively. Body composition measurements were obtained by using an octopolar (8 electrodes) bioelectrical impedance analyzer (BIA) (InBody 370, Seoul, Korea). The anthropometric variables such as skeletal muscle mass (SMM), fat mass, and BMI were evaluated. SMM is divided by body height squared to estimate the skeletal muscle mass index (SMI) (kg/m^2^), using the Janssen’s equation [24]. Appendicular skeletal muscle mass (ASM) is the total of the muscle mass in the arms and legs and served as a good substitute for whole-body skeletal muscle mass [25]. After a 5-minute rest, the brachial blood pressure was assessed using an automated blood pressure monitor (OMRON HEM-801 model).

#### Laboratory measurements

After a minimum 12-hours fast, venous blood was collected from participants. Biochemical indicators were measured using biochemical auto-analyzers (Mindray BS-430, Shenzhen, China). Moreover, a chemiluminescence immunoassay analyzer (Mindray CL-2000i, Shenzhen, China) was used to measure the levels of estradiol and the fasting serum 25-hydroxyvitamin D (25(OH) D) concentration. vitamin D deficiency was deemed to exist at a 25(OH) D concentration < 20 ng/mL (50nmol/L), and 25(OH) D concentrations ≥ 20 ng/mL (50nmol/L) were regarded as non-vitamin D deficiency [26].

### Definition of covariate

Low skeletal muscle mass has been referred to as skeletal muscle mass index (skeletal muscle mass/height^2^) < 5.7 kg/m^2^ in female [27]. The menopausal status of participants was divided into three groups: premenopausal (35 ~ 44 years), perimenopausal (45 ~ 54 years), and postmenopausal (≥ 55 years) [28]. Weight/height^2^ (kg/m^2^) was used to determine BMI [29]. The definitions of the smoking and alcohol consumption status were as follows: one cigarette per day for six consecutive months, and one drink per week for six consecutive months, respectively [30]. Primary school or below, junior high school, high school or above were the three divisions for the educational level. Physical activity (PA), which was rated as low, moderate, or high, was assessed using the International Physical Activity Questionnaire [31]. The dietary energy and nutrients of the participants were calculated through the Nutrition Calculator v2.8.0.

### Statistical analysis

Continuous variables with skewed distributions were presented as median (interquartile range, IQR) and as mean ± standard deviation (SD) for normal. Frequency (percentage) was used to express categorical variables. The Kruskal-Wallis test was used for continuous variables, and the chi-square test was applied to categorical variables. Bivariate logistic regression analyses for evaluating the effect of estradiol level and vitamin D status with low skeletal muscle mass. The combined impact of estradiol and low vitamin D status on low skeletal muscle mass was evaluated using multiplicative interaction. Menopausal status, residence, marital status, educational level, status of drinking and smoking, physical activity, triglyceride, glucose, and dyslipidemia status were included as covariates in the adjusted models. ORs and 95% CIs of low skeletal muscle mass in response to vitamin D deficiency across the quartiles of serum estradiol concentration were estimated, and their interactions were tested. The tolerance and variance inflation factor (VIF) of multicollinearity diagnosis were used to evaluate the validity of regression models. Additionally, we examined the trend of vitamin D quartiles in separate models by using ordinal values.

R 4.0.6 software was used to carry out the statistical analyses. A two-side *P* value < 0.05 was treated statistically significant.

## Results

### General characteristics

Table 1 displays the fundamental characteristics of the study participants. All of the 3025 participants, aged 56.27 ± 9.86 years. According to the skeletal muscle mass index, participants were split into two groups based on their levels of skeletal muscle mass: low skeletal muscle mass (low SMM) and normal skeletal muscle mass (normal SMM). A total of 287 (9.49%) participants were reported as low SMM, and had higher age than normal SMM. Moreover, there were notable differences between low and normal SMM in terms of marital status, educational background, and menopausal status (*P* < 0.001). In terms of anthropometric measurements, participants with low SMM had lower weight, height, BMI, WC, ASMI, and FM values than participants with normal SMM (*P* < 0.001). In terms of laboratory measurements, low SMM group had lower levels of triglycerides (TGs), estradiol and vitamin D concentration than normal SMM group (*P* < 0.05). Furthermore, participants with low SMM also had lower values of dietary energy, protein, fat and carbohydrate than with normal SMM (*P* < 0.001).

**Table 1:** General characteristics of study participants

Characteristics	Total	Low SMM (n = 287)	Normal SMM (n = 2738)	*P* value
Age (years)	56.27 ± 9.86	62.59 ± 9.54	55.60 ± 9.66	< 0.001
Marital status				
Married	2741 (90.6)	238 (82.9)	2503 (91.4)	< 0.001
Widowed	274 (9.1)	48 (16.7)	226 (8.3)	
Divorce and unmarried	10 (0.3)	1 (0.3)	9 (0.3)	
Education				
Primary school and below	2228 (73.7)	241 (84.0)	1987 (72.6)	< 0.001
Junior high school	714 (23.6)	40 (13.9)	674 (24.6)	
High school and above	83 (2.7)	6 (2.1)	77 (2.8)	
Weight (kg)	60.94 ± 9.19	48.88 ± 5.87	62.20 ± 8.53	< 0.001
Height (cm)	155.95 ± 5.96	151.27 ± 5.69	156.44 ± 5.77	< 0.001
BMI (kg/m^2^)	25.05 ± 3.45	21.42 ± 2.80	25.43 ± 3.28	< 0.001
WC (cm)	86.39 ± 9.10	77.46 ± 7.52	87.33 ± 8.74	< 0.001
ASMI (kg/m^2^)	6.58 ± 0.69	5.37 ± 0.30	6.71 ± 0.59	< 0.001
FM (kg)	21.80 ± 6.45	16.76 ± 5.43	22.33 ± 6.32	< 0.001
Cholesterol (mmol/L)	4.93 (4.30, 5.59)	4.95 (4.27, 5.63)	4.93 (4.30, 5.58)	0.925
HDL-C (mmol/L)	1.37 (1.18, 1.58)	1.39 (1.21, 1.63)	1.37 (1.18, 1.58)	0.211
LDL-C (mmol/L)	2.86 (2.35, 3.34)	2.77 (2.27, 3.37)	2.87 (2.35, 3.34)	0.446
Triglyceride (mmol/L)	1.48 (1.05, 2.11)	1.43 (0.99, 1.91)	1.49 (1.06, 2.13)	0.005
Glucose (mmol/L)	5.44 (4.98, 6.29)	5.48 (4.96, 6.73)	5.44 (4.98, 6.27)	0.315
Dyslipidemia (%)	1034 (34.2)	94 (32.8)	940 (34.3)	0.592
Estradiol Level (pg/mL)	14.81 (5.00, 33.94)	10.75 (5.00, 17.58)	15.38 (5.00, 36.21)	< 0.001
Vitamin D concentration (ng/ml)	12.07 (8.91, 15.74)	10.70 (8.14, 14.61)	12.20 (8.98, 15.81)	< 0.001
VD deficiency (%)	2724 (90.0)	263 (91.6)	2461 (89.9)	0.345
Menopausal Status (%)				
Pre-menopausal	408 (13.5)	15 (5.2)	393 (14.3)	< 0.001
Perimenopausal	1104 (36.5)	61 (21.3)	1043 (38.1)	
Postmenopausal	1513 (50.0)	211 (73.5)	1302 (47.6)	
Smoking (%)	36 (1.2)	5 (1.7)	31 (1.1)	0.365
Alcohol intake (%)	417 (13.8)	34 (11.8)	383 (14.0)	0.317
Physical activity (%)				
Low	946 (31.3)	85 (29.6)	861 (31.5)	0.817
Medium	1563 (51.6)	152 (53.0)	1411 (51.5)	
High	516 (17.1)	50 (17.4)	466 (17.0)	
Dietary energy (Kcal)	1645.63 (1113.80, 2190.93)	1456.63 (809.05, 2056.00)	1660.25 (1132.69, 2202.77)	0.002
Dietary protein (g)	59.66 (33.43, 85.88)	50.34 (22.41, 79.22)	60.92 (35.21, 86.58)	< 0.001
Dietary fat (g)	77.70 (50.73, 108.85)	70.63 (42.72, 102.84)	78.96 (52.06, 109.36)	0.001
Dietary carbohydrate (g)	206.27 (130.79, 331.39)	177.01 (100.79, 283.36)	209.21 (131.19, 334.93)	< 0.001

### Association between estradiol concentration and low skeletal muscle mass

A total of 287 (9.49%) participants with low SMM. Meanwhile, the participants with low estradiol concentrations also had a significantly higher risk of developing low SMM compared to those with normal estradiol concentrations (*P* < 0.001). Furthermore, the lowest to highest values of the estradiol concentrations were grouped into quartiles (Q1-Q4). The ranges of estradiol concentrations across quartiles were < 5.00, 5.00-<14.81, 14.81-<33.90, ≥ 33.90. As shown in Table 2. The odds of low skeletal muscle mass in estradiol concentration Q1, Q2, and Q3 were 2.354 (95% CI: 1.210–4.614), 2.882 (95% CI: 1.393–5.964), and 2.838 (95% CI: 1.419–5.676), respectively, compared to the odds in the highest quartile of estradiol concentration after adjusting for confounding variables in Model 3.

**Table 2:** The association between serum estradiol quartiles and low skeletal muscle mass

Quartiles of estradiol levels	Unadjusted Model			Model 1			Model 2			Model 3	
	OR (95%CI)	*P* value		OR (95%CI)	*P* value		OR (95%CI)	*P* value		OR (95%CI)	*P* value
Q1 (< 5.00)	4.724 (2.974, 7.504)	< 0.001		2.961 (1.683,5.208)	< 0.001		2.995 (1.701,5.272)	< 0.001		2.354 (1.210,4.614)	0.013
Q2 (5.00 ~ 14.81)	4.937 (2.991, 8.147)	< 0.001		3.219 (1.764,5.874)	< 0.001		3.248 (1.777,5.935)	< 0.001		2.882 (1.3935.964)	0.004
Q3 (14.81 ~ 33.90)	3.674 (2.259, 5.977)	< 0.001		2.808 (1.592,4.953)	< 0.001		2.847 (1.612,5.026)	< 0.001		2.838 (1.419,5.676)	0.003
Q4 (≥ 33.90)	Reference			Reference			Reference			Reference	
*P* for trend		< 0.001			< 0.001			< 0.001			< 0.001

Notes: The model 1 adjusted for age, residence, marital status, and educational level; Model 2 adjusted for model 1 and smoking status, drinking status, physical activity; Model 3 adjusted for model 2 and BMI, FM, triglyceride, menopausal status, dietary energy

### Relationship between vitamin D concentration and low skeletal muscle mass

As shown in Table 3. The serum vitamin D levels were also split into quartiles, which were < 8.91, 8.91-<12.07, 12.07-<15.74, ≥ 15.74, respectively. When compared to Q4, the adjusted ORs values for low skeletal muscle mass in vitamin D level Q1, Q2, and Q3 were 1.720 (95% CI: 1.201–2.674), 1.386 (95% CI: 0.892–2.154), and 1.142 (95% CI: 0.710–1.835), respectively, adjusted for confounding variables in Model 3.

**Table 3:** The association between serum Vitamin D quartiles and low skeletal muscle mass

Quartiles of vitamin D levels	Unadjusted Model			Model 1			Model 2			Model 3	
	OR (95%CI)	*P* value		OR (95%CI)	*P* value		OR (95%CI)	*P* value		OR (95%CI)	*P* value
Q1 (< 8.91)	1.617 (1.143, 2.287)	0.007		1.457 (1.016,2.090)	0.041		1.433 (1.005,2.071)	0.047		1.720 (1.201,2.674)	0.016
Q2 (8.91 ~ 12.07)	1.480 (1.041, 2.104)	0.029		1.281 (0.893,1.838)	0.178		1.272 (0.886,1.825)	0.192		1.386 (0.892,2.154)	0.146
Q3 (12.07 ~ 15.74)	0.959 (0.658, 1.404)	0.828		0.967 (0.655,1.427)	0.866		0.967 (0.655,1.428)	0.868		1.142 (0.710,1.835)	0.585
Q4 (≥ 15.74)	Reference			Reference			Reference			Reference	
*P* for trend		0.003			0.003			0.003			0.003

Notes: The model 1 adjusted for age, residence, marital status, and educational level; Model 2 adjusted for model 1 and smoking status, drinking status, physical activity; Model 3 adjusted for model 2 and BMI, FM, triglyceride, menopausal status, dietary energy

### The combined association of estradiol and vitamin D deficiency status on low SMM

**Fig. 1:**
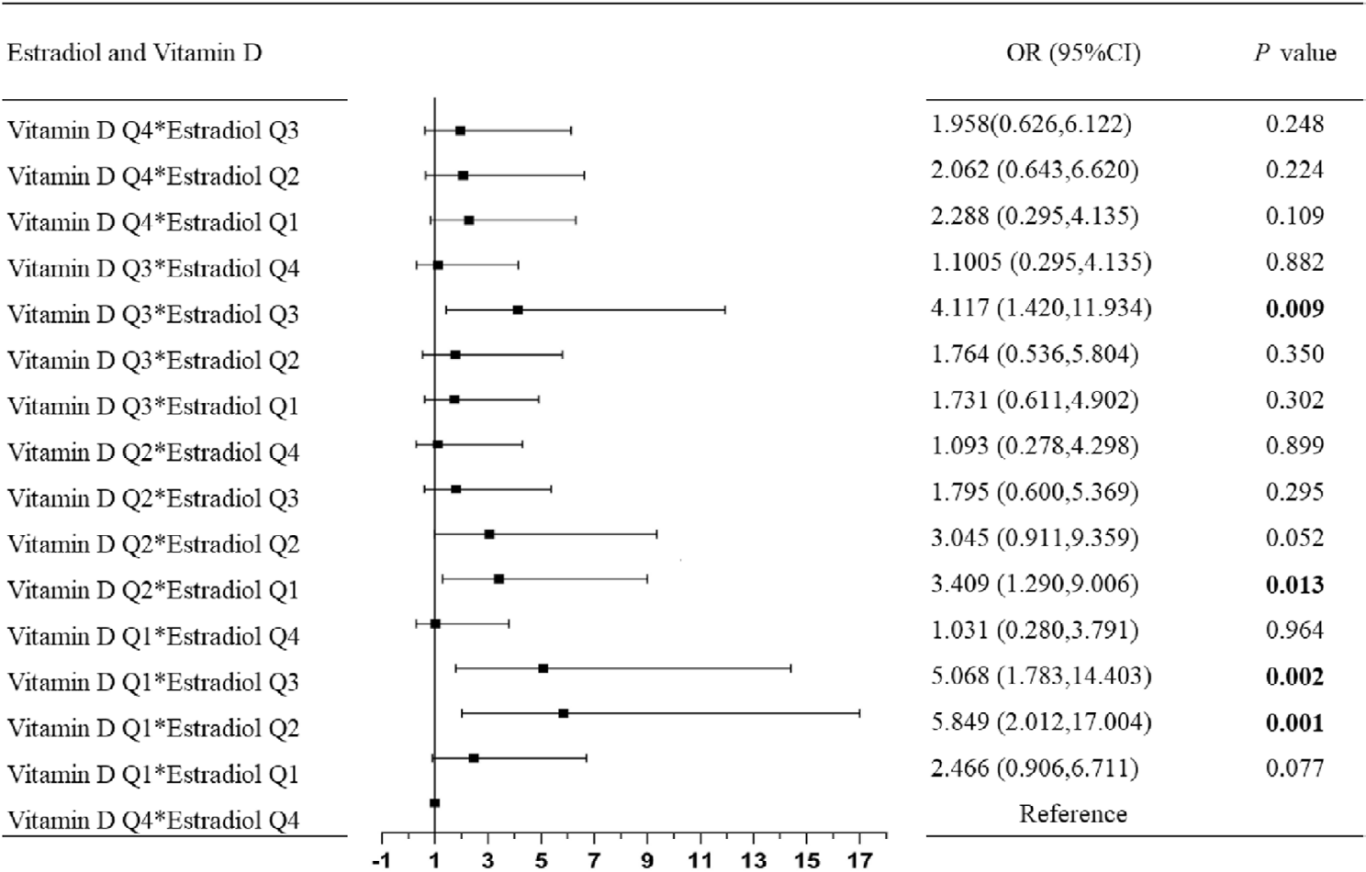
The forest plot of interactions between quartile of vitamin D and estradiol on low skeletal muscle mass. Adjusted for age, residence, marital status, educational level, smoking status, drinking status, physical activity, BMI, FM, triglyceride, menopausal status, and dietary energy. Presented the forest plot of interactions between quartile of vitamin D and estradiol on low skeletal muscle mass among middle-aged and elderly women. The significant interactions between vitamin D Q1 and estradiol Q2, vitamin D Q1 and estradiol Q3, vitamin D Q2 and estradiol Q1, vitamin D Q3 and estradiol Q3 were stably reflected in the final model (P for interaction < 0.05), after adjusting potential confounding factors

Participants with the lower quartile of estradiol concentrations and vitamin D deficiency had a higher likelihood of developing low skeletal muscle, compared to those in the highest quartile of estradiol concentrations and non-vitamin D deficiency (Table 4). Considering the applicability of the definition of vitamin D deficiency in the population, we further used the vitamin D quartile to explore the interaction between vitamin D and estradiol on low SMM. The significant interactions between vitamin D Q1 and estradiol Q2, vitamin D Q1 and estradiol Q3, vitamin D Q2 and estradiol Q1, vitamin D Q3 and estradiol Q3 were stably reflected in the three models (*P* for interaction < 0.05; Table 5; Fig. 1).

**Table 4:** Odds ratios for the combined association of estradiol quartiles and vitamin D deficiency status with low SMM.

Estradiol and Vitamin D deficiency	Unadjusted Model			Model 1			Model 2			Model 3	
	OR (95%CI)	*P* value		OR (95%CI)	*P* value		OR (95%CI)	*P* value		OR (95%CI)	*P* value
Q1 + VD deficiency	3.849 (1.393, 10.634)	**0.009**		2.776 (0.985,7.824)	0.053		2.879 (1.021,8.123)	**0.046**		3.898 (1.155,13.160)	**0.028**
Q2 + VD deficiency	3.921 (1.391, 11.059)	**0.010**		2.946 (1.021,8.502)	**0.046**		3.047 (1.054,8.802)	**0.040**		4.801 (1.378,16.721)	**0.014**
Q3 + VD deficiency	2.987 (1.068, 8.355)	**0.037**		2.628 (0.924,7.479)	0.070		2.738 (0.961,7.8025)	0.059		4.709 (1.365,16.251)	**0.014**
Q4 + VD deficiency	0.768 (0.255, 2.313)	0.639		0.910 (0.295,2.807)	0.870		0.941 (0.304,2.910)	0.916		1.970 (0.527,7.365)	0.314
Q1 + non-VD deficiency	3.057 (0.887, 10.538)	0.077		2.389 (0.679,8.405)	0.175		2.530(0.717,8.921)	0.149		3.416 (0.766,15.237)	0.107
Q2 + non-VD deficiency	4.338 (1.160, 16.226)	**0.029**		3.437 (0.898,13.148)	0.071		3.637 (0.948,13.955)	0.060		3.948 (0.745,20.935)	0.107
Q3 + non-VD deficiency	2.548 (0.692, 9.375)	0.159		2.368 (0.631,8.891)	0.201		2.488 (0.650,9.217)	0.186		4.454 (0.914,21.711)	0.065
Q4 + non-VD deficiency	Reference			Reference			Reference			Reference	

Notes: Bold indicates statistical significance. The model 1 adjusted for age, residence, marital status, educational level, BMI and fat mass; Model 2 adjusted for model 1 and smoking status, drinking status, physical activity; Model 3 adjusted for model 2 and triglyceride, menopausal status, dietary energy

**Table 5:** Odds ratios for the combined association of estradiol quartiles and vitamin D quartiles with low SMM.

Vitamin D and Estradiol	Unadjusted Model			Model 1			Model 2			Model 3	
	OR (95%CI)	*P* value		OR (95%CI)	*P* value		OR (95%CI)	*P* value		OR (95%CI)	*P* value
VD Q1 + estradiol Q1	3.339 (1.506,7.404)	**0.003**		2.140 (0.920,4.982)	0.077		2.178 (0.936,5.073)	0.071		2.466 (0.906,6.711)	0.077
VD Q1 + estradiol Q2	7.008 (3.061,6.048)	**< 0.001**		4.915 (2.035,11.871)	**< 0.001**		4.964 (2.054,11.996)	**< 0.001**		5.849 (2.012,17.004)	**0.001**
VD Q1 + estradiol Q3	4.818 (2.128,10.907)	**< 0.001**		3.651 (1.545,8.629)	**0.003**		3.704 (1.566,8.758)	**0.003**		5.068 (1.783,14.403)	**0.002**
VD Q1 + estradiol Q4	0.723 (0.232,2.249)	0.575		0.756 (0.239,2.391)	0.634		0.751 (0.237,2.377)	0.626		1.031 (0.280,3.791)	0.964
VD Q2 + estradiol Q1	5.154 (2.387,11.130)	**< 0.001**		3.35 (1.485,7.556)	**0.004**		3.393 (1.503,7.659)	**0.003**		3.409 (1.290,9.006)	**0.013**
VD Q2 + estradiol Q2	4.452 (1.841,0.767)	**0.001**		2.731 (1.078,6.918)	**0.034**		2.742 (1.081,6.955)	**0.034**		3.045 (0.911,9.359)	0.052
VD Q2 + estradiol Q3	2.058 (0.844,5.020)	0.113		1.466 (0.579,3.712)	0.419		1.493 (0.59,3.781)	0.398		1.795 (0.600,5.369)	0.295
VD Q2 + estradiol Q4	0.655 (0.194,2.214)	0.496		0.707 (0.208,2.410)	0.580		0.715 (0.210,2.441)	0.593		1.093 (0.278,4.298)	0.899
VD Q3 + estradiol Q1	2.703 (1.170,6.244)	**0.020**		1.856 (0.772,4.458)	0.167		1.879 (0.782,4.518)	0.159		1.731 (0.611,4.902)	0.302
VD Q3 + estradiol Q2	1.711 (0.626,4.675)	0.295		1.216 (0.429,3.449)	0.714		1.243 (0.438,3.527)	0.683		1.764 (0.536,5.804)	0.350
VD Q3 + estradiol Q3	3.012 (1.310,6.927)	**0.009**		2.59 (1.096,6.121)	**0.030**		2.662 (1.125,6.298)	**0.026**		4.117 (1.420,11.934)	**0.009**
VD Q3 + estradiol Q4	0.681 (0.219,2.118)	0.507		0.713 (0.227,2.237)	0.563		0.731 (0.232,2.297)	0.591		1.101 (0.295,4.135)	0.882
VD Q4 + estradiol Q1	3.219 (1.424,7.275)	**0.005**		2.183 (0.926,5.147)	0.074		2.256 (0.956,5.324)	0.063		2.288 (0.295,4.135)	0.109
VD Q4 + estradiol Q2	2.849 (1.130,7.178)	**0.026**		2.144 (0.818,5.615)	0.121		2.191 (0.835,5.751)	0.111		2.062 (0.643,6.620)	0.224
VD Q4 + estradiol Q3	1.816 (0.726,4.544)	0.202		1.685 (0.654,4.339)	0.280		1.721 (0.668,4.436)	0.261		1.958 (0.626,6.122)	0.248
VD Q4 + estradiol Q4	Reference			Reference			Reference			Reference	

Notes: Bold indicates statistical significance. The model 1 adjusted for age, residence, marital status, educational level, BMI and fat mass; Model 2 adjusted for model 1 and smoking status, drinking status, physical activity; Model 3 adjusted for model 2 and triglyceride, menopausal status, dietary energy

## Discussion

The primary objective of this study was to determine whether concentrations of vitamin D and estradiol were related to low SMM among middle-aged and elderly women. Our study revealed a negative correlation between low skeletal muscle mass and levels of vitamin D and estradiol. However, when considered confounding factors, including age, residence, marital status, educational level, smoking and drinking status, physical activity, BMI, FM, triglyceride, menopausal status, and dietary energy, these correlations were maintained in estradiol Q1, Q2, Q3 and vitamin D Q1. Furthermore, we evaluated whether there was any combined association of estradiol and vitamin D levels on low SMM. In this respects, we found that participants with the lower quartile of estradiol concentrations and vitamin D deficiency had a higher likelihood of developing low skeletal muscle, compared to those in the highest quartile of estradiol concentrations and non-vitamin D deficiency. Considering the applicability of the definition of vitamin D deficiency in the population, we further used the quartile of vitamin D to explore the interaction between vitamin D and estradiol on low SMM, obtaining similar results.

To our knowledge, not only is the connection between muscle atrophy and vitamin D and estradiol status ambiguous, but also these combined effects on muscle atrophy have not been studied. This study is the first study evaluating the combined association of estradiol and vitamin D levels on low SMM among middle-aged and elderly women. Previous studies have found that the muscle atrophy onset is multifactorial and determined by several promoting factors [32, 33]. Menopause seems to be particularly closely correlated with the beginning of muscle atrophy in women, which brings about hormone changes, especially estradiol [34]. Besides, muscle atrophy may also be triggered by vitamin D [35].

While estradiol levels change throughout life, the majority of women experience the menopausal transition in their mid-40s, a period of time during which the level of female hormones decreases and the menstrual cycle becomes irregular. In fact, estradiol is crucial for preserving muscle health in older people [36]. Previous researches have shown a close relationship between estradiol concentrations decrease and muscle atrophy [37, 38], which is in line with the findings of this investigation. However, controversial results have been reported, indicating that estradiol has no impact on postmenopausal women’s muscle atrophy [39]. Despite all this, several basic experimental studies have confirmed that interleukin (IL)-6, IL-1, and tumor necrosis factor-a (TNF-α) production are all closely associated with menopause [40]. Meanwhile, the IL-6 and TNF-α can impair muscle performance [41, 42], causing sarcopenia [43, 44].

Additionally, vitamin D, a vital hormone linked to muscle physiology, appears to be involved in muscle atrophy [45]. According to prior researches, low muscle strength and physical performance of elderly adults are associated with their low vitamin D concentration [46–49]. Meanwhile, other studies also revealed that older adults with vitamin D deficiency are more likely to develop muscle mass and muscle strength decline [50, 51]. These outcomes are consistent with those from our study, which demonstrated a significant relationship between low vitamin D concentrations and low skeletal muscle mass in relation to recent muscle atrophy. Given the lack of clearly defined mechanisms, the genomic pathway using the nuclear receptor VDR [52] and the non-genomic pathway, where vitamin D enters and affects cells directly through the cell surface fossa [53], are the potential mechanisms. Myofibres and numerous other cell types combine to form the mature skeletal muscle, which also contains vitamin D target cells [54]. As a result, vitamin D may interact intricately with the different skeletal muscle cells and tissues to affect muscle mass and performance [35].

In this study, low concentrations of vitamin D and estradiol have combined effect on muscle atrophy. Estradiol is known to decrease among the menopausal transition and vitamin D deficiency occur at lots of countries worldwide [55]. According to prior research, the relationship between vitamin D and estradiol in female populations remains inconclusive. Some studies have found a significant negative correlation between 25(OH) D and estrogen in fertile women [56], while similar findings were observed in postmenopausal women [57]. On the other hand, other studies have reported a positive correlation between vitamin D and estradiol [58], suggesting that their relationship may be influenced by multiple factors and may involve mutual regulation. Meanwhile, the hormone as a crucial element throughout life and decrease with aging, especially in muscle atrophy development. Notably, other evidences suggested that the hormone replacement therapy is good for muscle mass and strength in postmenopausal women [59, 60]. Nutritional and lifestyle factors may not only benefit muscle mass and performance, but also affordable and secure. However, despite the fact that a lot of research has been done in the past on supplement therapy, more research is required to evaluate the effectiveness and long-term safety of potential therapeutic interventions aimed at improving muscle function.

The present study has several benefits, including the fact that potential confounding factors like socioeconomic status, lifestyle, and macronutrients were taken into account when conducting the analyses. Moreover, from a dietary and nutritional perspective, it should also be pointed out that our research design and analysis methodology can effectively prevented confounding changes in overall macronutrient balance and dietary intake. Simultaneously, in contrast to prior research, this study also explored the impact of low skeletal muscle mass in middle-aged and elderly women, examining the interaction between estradiol and vitamin D. However, constraints on the current study should be taken into account. First, instead of dual-energy X-ray absorptiometry, which is the gold standard method for determining human body composition, BIA was employed because it is a reliable and useful method [61]. Second, we have to emphasize that a relatively small sample of rural women were included in this research, so the more in-depth research is still needed.

## Conclusions

In conclusion, vitamin D deficiency is common among middle-aged and elderly women, and estradiol declines during the menopausal transition, both of which have important fundamental biological effects and interactions on muscle mass and performance. We conclude the vitamin D and estradiol were interactively correlated with low skeletal muscle mass in middle-aged and elderly women. Therefore, the combination of estradiol and vitamin D supplements should be encouraged for middle-aged and elderly women who are at risk of muscle atrophy or experiencing muscle atrophy.

## Data Availability

The datasets generated and analyzed during the current study are not publicly available due to privacy or ethical restrictions but are available from the corresponding author on reasonable request.
