# Correction: Interaction of estradiol and vitamin D with low skeletal muscle mass among middle-aged and elderly women

**DOI:** 10.1186/s12905-023-02687-4

**Published:** 2023-10-24

**Authors:** Jiaxing Zhang, Yalong Cheng, Chen Chen, Qingan Wang, Chan Yang, Jiangwei Qiu, Juan Li, Xiaowei Liu, Yuhong Zhang, Lan Liu, Yi Zhao

**Affiliations:** 1 School of Public Health, Ningxia Medical University, Ningxia Hui Autonomous Region, Yinchuan, China; 2 Department of Public Health, People’s Hospital of Ningxia Hui Autonomous Region, Yinchuan, Ningxia China; 3 Key Laboratory of Environmental Factors and Chronic Disease Control, Ningxia Medical University, Yinchuan, Ningxia China; 4 School of Nursing, Ningxia Medical University, Ningxia Hui Autonomous Region, Yinchuan, China

**Correction to: BMC Women’s Health (2023) 23:491**

10.1186/s12905-023-02646-z

In this article [1], “Lan Liu (liulan-162@163.com)” should have been denoted as a corresponding author.

Under funding information section, a funder name and an institution “Natural Science Foundation of Ningxia Province (2022AAC03143)” needs to be added. So the Funding information should read as “The research was supported by National Key Research and Development Program of China (2017YFC0907204), the Key Research and Development Program of Ningxia (2021BEG02026) and Natural Science Foundation of Ningxia Province (2022AAC03143)”.

The original article has been corrected.
